# The interplay between the tumor microenvironment and tumor-derived small extracellular vesicles in cancer development and therapeutic response

**DOI:** 10.1080/15384047.2024.2356831

**Published:** 2024-05-20

**Authors:** Xuanyu Guo, Jiajun Song, Miao Liu, Xinyi Ou, Yongcan Guo

**Affiliations:** a The Affiliated Hospital, Southwest Medical University, Luzhou, PR China; b Department of Clinical Laboratory Medicine, the Affiliated Hospital, Southwest Medical University, Luzhou, PR China; c Nanobiosensing and Microfluidic Point-of-Care Testing, Key Laboratory of Luzhou, Department of Clinical Laboratory, The Affiliated Traditional Chinese Medicine Hospital, Southwest Medical University, Luzhou, PR China

**Keywords:** Tumor microenvironment, tumor-derived small extracellular vesicles, hypoxic microenvironment, acidic microenvironment, immunosuppressive microenvironment, targeted therapy

## Abstract

The tumor microenvironment (TME) plays an essential role in tumor cell survival by profoundly influencing their proliferation, metastasis, immune evasion, and resistance to treatment. Extracellular vesicles (EVs) are small particles released by all cell types and often reflect the state of their parental cells and modulate other cells’ functions through the various cargo they transport. Tumor-derived small EVs (TDSEVs) can transport specific proteins, nucleic acids and lipids tailored to propagate tumor signals and establish a favorable TME. Thus, the TME’s biological characteristics can affect TDSEV heterogeneity, and this interplay can amplify tumor growth, dissemination, and resistance to therapy. This review discusses the interplay between TME and TDSEVs based on their biological characteristics and summarizes strategies for targeting cancer cells. Additionally, it reviews the current issues and challenges in this field to offer fresh insights into comprehending tumor development mechanisms and exploring innovative clinical applications.

## 1. Introduction

**Figure 1.**
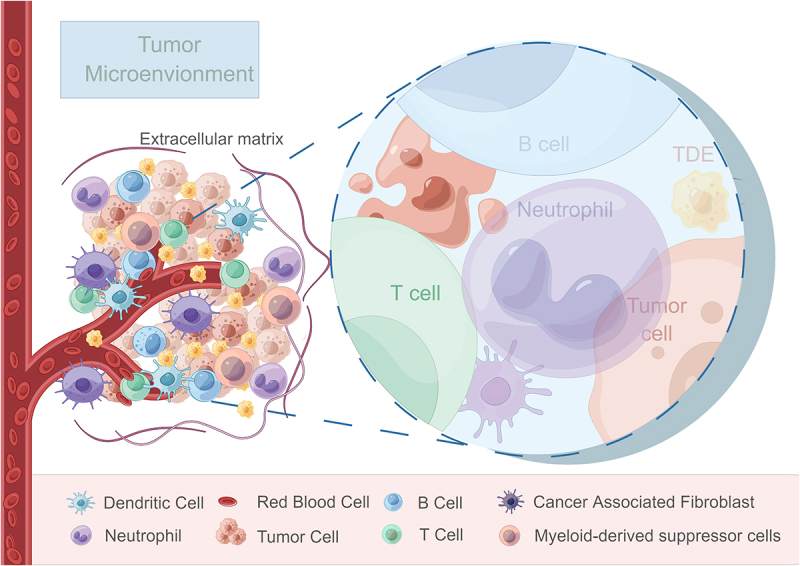
Schematic representation illustrating the components and interactions within the tumor microenvironment.

The tumor microenvironment (TME) comprises a complex interplay of physiological and biochemical elements, including tumor cells, tumor-associated fibroblasts, innate and adaptive immune cells, the vascular system, cytokines, and the extracellular matrix (ECM) network^1^ (Figure 1). Changes within the TME significantly influence tumor growth, invasion, metastasis and the effectiveness of therapeutic interventions and often arise due to factors such as primary organ location, tumor characteristics, stage and patient status, which can then lead to consequential impact on drug resistance and treatment outcomes.^2^ Recent studies have also confirmed the interaction between tumor cells and tumor-associated immune cells in TME, reporting the effects of tumor progression and metastasis through different signal transduction or receptor activation pathways.^3^ The TME can be characterized by hypoxia,^4^ acidity,^5^ nutrient deprivation^6^ and immunosuppression^7^ due to the rapid tumor growth, increased swelling, and inadequate vascularization. These unique features provide a favorable microenvironment for tumor development and dissemination.

Extracellular vesicles (EVs) are membrane-bound structures present in various bodily fluids, including blood, urine, and ascites. Among these, small extracellular vesicles (sEVs), typically measuring less than 200 nm in diameter, fulfill diverse physiological and pathological functions.^8^

Tumor-derived small extracellular vesicles (TDSEVs) are continuously released by tumor cells and serve as specialized signaling carriers within theTME.^9^ As an important form of communication between tumor cells and non-tumor cells in the microenvironment, TDSEVs play essential roles in various stages of tumor development.^10^ Recent findings indicate that TDSEVs harbor a diverse array of molecular constituents, including lipids, membrane-associated proteins, long noncoding RNAs (lncRNAs), and miRNAs,^10^ which contribute to key processes in tumor initiation and progression, such as angiogenesis, proliferation, metastasis, and immune evasion.^11^ Moreover, the prominent biological features of TME, such as hypoxia, extracellular acidosis and low nutrients, can induce heterogeneous changes in TDSEVs release, cargo composition and transport, which are also key determinants for malignancy, proliferation, and metastasis.^9,12,13^ Therefore, it is imperative not to overlook the role of TDSEVs in maintaining the survival environment of tumor cells and promoting their invasion and metastasis. Investigating the mechanisms by which TDSEVs mediate tumor occurrence and development through their interaction with the TME could offer valuable guidance for devising more effective tumor prognosis and treatment strategies based on TDSEVs and TME interactions.

This review examines the interaction between TMEs and TDSEVs, with a primary focus on the biological characteristics of TMEs. We focus on the reciprocal regulatory mechanisms of TDSEVs within tumor hypoxic, acidic, nutrient-deprived and immunosuppressive microenvironments and discuss targeted therapeutic approaches involving TDSEVs or TMEs, along with the predominant challenges currently faced in this field. Additionally, we provide a comprehensive review of the potential of targeted strategies directed toward TMEs and TDSEVs in tumor therapy to provide insights into potential clinical treatment methods and present innovative concepts for combating tumors.

## 2. Generation and characteristics of EVs

### 2.1. EVs and exosomes

**Figure 2.**
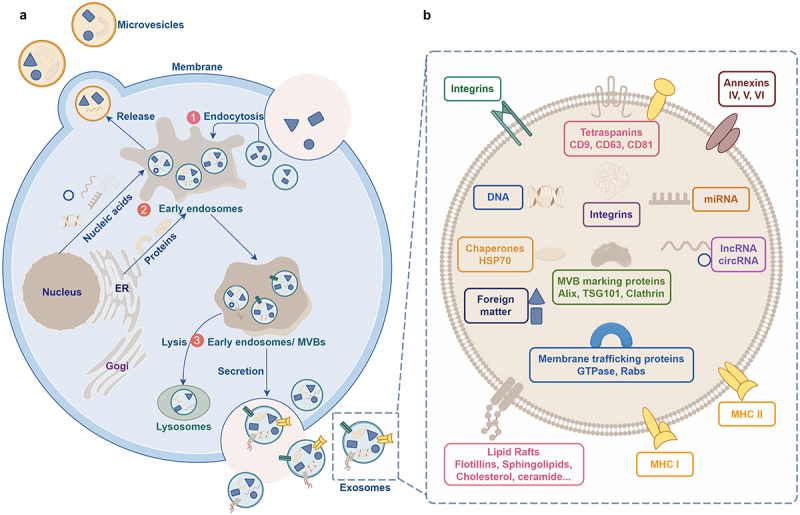
Schematic representation depicting the process of exosome biogenesis and release. A. The steps involved in exosome biogenesis: ① Invagination of the plasma membrane. ② Formation of early endosomes. ③ Maturation of early endosomes into late endosomes, which develop into multivesicular bodies (MVBs). The membrane invagination of MVBs results in the formation of intraluminal vesicles (ILVs), which then fuse with the plasma membrane and release ILVs as exosomes. B. Schematic representation illustrating the structure of exosomes.

EVs are membranous particles released from cells, bounded by lipid bilayers, and lacking a functional nucleus, thus incapable of self-replication.^8^ Exosomes are a subtype of EVs secreted by cells. They can be broadly categorized into ectosomes and exosomes.^14^ Exosomes typically range from 30 to 150 nm (with a mean of 100 nm) in size and are formed via the endocytic pathway through several sequential steps.^14^ Initially, plasma membrane invagination leads to the formation of early endosomes.^15,16^ Subsequently, intracellular multivesicular bodies (MVBs) arise through inward budding, generating intraluminal vesicles (ILVs) enveloped within endosomes.^16^ Ultimately, ILVs fuse with the cell membrane, facilitating exosome release^16^ (Figure 2). The size of MVBs ranges between 100 and 250 nm, and they harbor multiple ILVs, which themselves vary in diameter from 30 to 150 nm.^17^ Various proteins participate in ILV and MVB formation, as well as cargo selection. Notably, endosomal sorting complexes required for transport (ESCRT) proteins, such as ESCRT-0, -I, -II and -III,^16,18^ are important in exosome biogenesis and possess distinct roles in membrane shaping and cargo sorting, wherein ESCRT-0 and ESCRT-I facilitate the association of ubiquitinated cargo with lipid microdomains, while ESCRT-II and -III mediate invagination and MVB/ILV formation.^9^ Additionally, an ESCRT-independent pathway exists for exosome biogenesis.^19^

Ectosomes are vesicles that bud off from the plasma membrane via outward budding and encompass microvesicles, microparticles, and larger vesicles ranging from 50 to 1000 nm in diameter.^14^ Despite the considerable knowledge regarding exosomes, our understanding on the biosynthesis of ectosomes remains largely unclear. The process of ectosome formation requires the accumulation of cargo on specific microdomains of the cytosolic surface of the plasma membrane. Concurrent membrane dynamics involve outward budding and fission within these microdomains. It is believed that the rearrangement of asymmetric membrane phospholipid layers, mediated by Ca^2+^-dependent enzymes, flippases and floppases may contribute to this process.^20^ Notably, the activation of at least two ESCRT complexes is similar to the mechanism observed during intraluminal vesicle (ILV) formation.^21^ Other mechanisms that regulate ectosome shedding from the plasma membrane include Arf6, a small GTPase implicated in vesicular transport, and members of the Rho family of GTPases, such as RhoA, Cdc42, and Rac1, which facilitate cortical actin contraction beneath the plasma membrane.^22–25^ Furthermore, ectosome production typically occurs across a large, extensive portion of the plasma membrane in response to various stimuli. Similar to exosomes, ectosomes also exhibit high levels of cholesterol, sphingomyelin, and ceramides.^26^

However, there is currently no consensus on specific biomarkers for EV subtypes, making it challenging to categorize EVs into distinct biogenesis pathways. Consequently, much of the existing literature on “exosomes” and “ectosomes/microvesicles” addresses heterogeneous populations of EVs rather than those released via specific biogenesis routes.^8^ To address this ambiguity, the International Society for Extracellular Vesicles (ISEV) emphasizes careful characterization, often relying on particle diameter to define EV subtypes.^8^ Specifically, sEVs are typically described as <200 nm in diameter, while large EVs exceed 200 nm in diameter.^8^ Exosomes constitute a subset of sEVs, with endosomes typically containing intracellular vesicles smaller than 200 nm in diameter. Ectosomes can vary in size, including those resembling exosomes. The sEV population encompasses both exosomes and small ectosomes. Henceforth, this review will consistently use sEVs to denote the term “exosome” in the literature.

### 2.2. TDSEVs

TDSEVs are sEVs released by tumor cells and are commonly detected in tumor tissue as well as in the body fluids of cancer patients.^27,28^ The biogenesis process of TDSEVs is similar to that of normal cells, yet tumor cells employ various strategies to modulate sEV biogenesis mechanisms, thereby facilitating tumor progression.^29^ Notably, tumor cells produce and secrete significantly greater amounts of sEVs compared to normal proliferating cells, with elevated sEV levels frequently observed in the plasma and other bodily fluids of cancer patients.^30–35^ Factors such as the hypoxic conditions prevalent in the TME have been reported to contribute to this robust sEV secretion by tumor cells.^36^ Furthermore, certain enzymes or proteins play roles in regulating sEV production from tumor cells. For instance, the p53 protein, often aberrantly activated in cancer, is implicated in regulating sEV production and release by tumor cells.^37^ Heparanase, overexpressed in many tumor cell lines, also regulates sEV secretion.^38^ Moreover, Rap GTPase proteins, particularly Rab27a and Rab27b, which regulate secretory pathways, are strongly associated with sEV release.^39,40^ The knockdown of Rab proteins has been shown to reduce sEV secretion from tumor cells.^40,41^ Despite emerging insights into the regulation of sEV secretion by tumor cells, the precise mechanisms remain unclear. In the TME, TDSEVs are essential for intercellular communication, transferring messages from the parent tumor cell to recipient cells and modulating autocrine, juxtacrine, and paracrine signaling pathways essential for cancer cell survival.^42^ Importantly, the paracrine activity of TDSEVs extends beyond the tumor site, as they can circulate and convey information to distant tissues and cells.^43^ Recent studies have demonstrated that TDSEVs can promote the activation, expansion, and immunosuppressive function of immune cells.^44^ They also participate in tumor progression and immune regulation and mediate intercellular communication within the TME.

## 3. Interplay between TMEs and TDSEVs

### 3.1. Hypoxic TME

A hypoxic TME is associated with increased intercellular communication, characterized by greater frequency and complexity of signal transduction. Emerging evidence indicates that sEVs, functioning as signaling transporters, are involved in the intricate process of hypoxia-induced signaling.^45,46^

#### 3.1.1. Affection of TDSEVs secretion and heterogeneity

**Figure 3.**
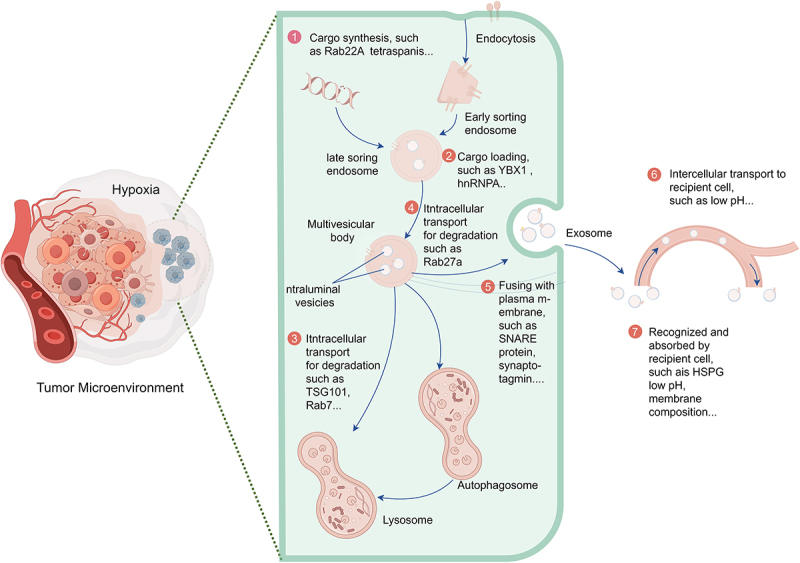
Schematic diagram illustrating key steps in the biogenesis and transport of tumor-derived exosomes in a hypoxic tumor microenvironment. ① Hypoxia influences cargo synthesis and sorting within exosomes. ② Hypoxia affects cargo loading into exosomes. ③ Hypoxia regulates exosome biogenesis by influencing the degradation of transporters (such as TSG101). ④ the hypoxic environment regulates exosome release by influencing the degradation of Rab GTPases (such as Rab27a). ⑤ the hypoxic environment regulates exosome release by affecting SNARE complexes specifically required for multivesicular body (MVB) fusion with the plasma membrane. ⑥ the hypoxic microenvironment influences the transport of tumor-derived exosomes to recipient cells. ⑦ the hypoxic microenvironment affects the recognition and uptake of tumor-derived exosomes by recipient cells.

Firstly, there is a significant increase in sEV release from the hypoxic microenvironment. Cancer cells respond to hypoxia by producing more sEVs, thus fulfilling the communication requirements of cancer cells compared to the normoxic state. This phenomenon has been observed in diverse solid tumor types, including breast cancer,^36,47^ colorectal cancer (CRC),^48,49^ glioma,^50^ gastric cancer,^51^ hepatocellular carcinoma (HCC),^52^ and pancreatic cancer.^53^ However, Ramteke et al. found that prostate cancer cells (LNCaP) secreted a comparable amount of sEVs under normoxic and hypoxic conditions (0.26E8 vs. 0.24E8 particles/mL).^54^ Additionally, hypoxia has been found to increase the number of sEVs in non-tumor cells, indicating the widespread occurrence of hypoxia-induced sEV release.^55–57^ Currently, it is widely accepted that hypoxia affects the release of sEVs through three main mechanisms. First, hypoxia affects key phases in sEV release, including cargo sorting, plasma membrane fusion,^58^ and MVB transport.^58,59^ The hypoxic TME impacts the biogenesis and release of TDSEVs, as illustrated in Figure 3. Second, hypoxia triggers certain releases by involving cofactors such as actin cytoskeletons,^60^ hypoxia regulators,^56^ microtubules,^61^ and molecular motors.^62^ Third, hypoxia’s involvement in the degradation process of MVBs leads to the release of intraluminal vesicles (ILVs) as exosomes.^63^ However, the specific effects of hypoxia and interactions with these key molecules remain largely unexplored.

Notably, hyperoxia has been observed to diminish the release of sEVs from CRC cells compared to hypoxia.^48^ Moreover, hypoxia can augment the heterogeneity of TDSEVs, which can be characterized by variations in size, cargo composition, and functional effects on recipient cells.^14^ Empirical evidence in the literature suggests that hypoxia induces the release of smaller EVs, as demonstrated in studies on various tumor cell lines, including CRC,^49,64^ pancreatic cancer,^53^ prostate cancer,^54^ and bone marrow mesenchymal stem cells.^65^ Additionally, hypoxia influences the cargo-sorting mechanism of TDSEVs.^66–69^ Similarly, the composition of EV cargo, encompassing glycoconjugates, lipids, nucleic acids, and proteins, exhibits variability among sEVs derived from different cancer types. While the specific effects of hypoxia on different cancer cells may vary or remain unclear, hypoxia profoundly impacts the quantity, composition, and status of EV cargo. This influence extends to cargo loading in cancer cells through intricate sorting mechanisms. Investigating the precise mechanisms underlying EV cargo loading in hypoxic cancer cells promises significant advancements in developing sEV-based interventions tailored for the hypoxic TME, thereby enhancing cancer therapy.

#### 3.1.2. Promoting TME remodeling

Hypoxia-induced TDSEVs possess the potential to promote tumor angiogenesis and metastasis. Recent studies have validated that hypoxic HCC-derived sEVs containing miR-1273f and miR-23a/b enhance the invasive phenotype of cancer cells, contributing to HCC progression.^70,71^ Additionally, Xue et al. demonstrated that sEVs derived from hypoxic bladder cancer (BCa) cells exhibit a greater capacity to stimulate BCa proliferation compared to those from normal BCa cells.^72^ Moreover, TDSEVs induced by hypoxia influence cancer immune evasion. Accumulating evidence suggests that hypoxia-induced TDSEVs can induce T cell apoptosis, suppress natural killer (NK) cell activity, inhibit type II macrophage expression dependent on IFN-γ, alter monocyte differentiation, and increase the population of myeloid-derived suppressor cells (MSDCs).^72–74^ Consequently, these immune response alterations lead to diminished immune surveillance and facilitate tumor evasion from immune recognition.

In summary, TDSEVs in hypoxic TME exhibit multifaceted functions and variations, emphasizing the importance of further exploration in this area to accurately delineate the TME landscape. Advancements in single-cell transcriptomic and spatial transcriptomic strategies could provide both challenges and opportunities for investigating different temporal, spatial, and subpopulations of sEVs within the hypoxic TME.

### 3.2. Acidic TME

The TME is characterized by altered tumor cell metabolism, resulting in an acidic pH, which stems from abnormal cell-cell interactions and disrupted homeostasis. In this metabolic state, tumor cells primarily rely on glycolysis over oxidative phosphorylation for energy production – a phenomenon known as anaerobic glycolysis. Consequently, there is a significant increase in lactate levels in the extracellular environment, accompanied by the diffusion of H^+^ ions into the cell stroma, ultimately leading to acidification of the extracellular pH within the TME.^75^

#### 3.2.1. Affecting the properties of TDSEVs

An acidic TME promotes the secretion of sEVs and facilitates their subsequent uptake and fusion with cells. Research has shown a significant increase in the levels of proteins, nucleic acids, and surface biomarkers in tumor-derived sEVs within an acidic environment, while these markers were not detected in an alkaline environment.^76^ In vitro studies have demonstrated a notable increase in sEV secretion when the pH is shifted from 7.4 to the characteristic acidic pH of 6.5 found in cancer cells, highlighting the important significance of tumor acidity in promoting heightened sEV release in human cancer cell lines.^12,77,78^ Parolini et al. reported that sEV release is enhanced under low pH conditions, accompanied by an increase in the content of sphingomyelin/ganglioside GM3 within sEVs, thereby promoting their uptake and fusion with recipient cells.^77^ Recent investigations have revealed that cells experiencing similar conditions within the TME (i.e., low pH and hypoxia) show a notable increase in the uptake of tumor-derived sEVs by parental cells, particularly under low pH treatment.^79^ The self-aggregation of glycerolipids between tumor cells and their sEVs has been identified as the underlying cause of the increased homologous uptake of TDSEVs.^79^ Moreover, Hisey et al. quantified sEV release from ovarian cancer cells using nanoparticle tracking analysis (NTA) combined with nanoscale flow cytometry (NFC) and provided experimental evidence for the essential role of TME pH in regulating sEV production and release.^80^ Therefore, alkalinization of the TME may hold promise as a strategy for the treatment of various cancer diseases.

#### 3.2.2. Promoting tumor progression

The release of TDSEVs in response to an acidic TME contributes significantly to invasion and metastasis. In an acidic TME, TDSEVs facilitate communication between cancer cells and stromal cells, thereby driving the formation of pre-metastatic niches (PMNs) and promoting metastasis and resulting in the acquisition of metastatic properties within the primary tumor or at distant sites.^41^ Tian et al. demonstrated that an acidic TME upregulated the expression levels of miR-21 and miR-10b in HCC-derived sEVs,^81^ which promoted the migration and invasion of recipient HCC cells cultured under normal conditions, both in vitro and in vivo.^81^ Furthermore, the presence of an acidic TME and the associated up-regulation of miR-21 and miR-10b in sEVs have been correlated with poor prognosis for HCC, highlighting the essential role of acidic TME-induced TDSEVs in invasion and metastasis.^81^

Furthermore, TDSEVs regulate tumor angiogenesis through multiple pathways and targets by transporting and delivering various tumor angiogenic factors within the acidic TME. He et al. discovered that sEV miR-205 secreted by ovarian cancer cells induces tumor angiogenesis by modulating the PTEN-AKT signaling pathway.^82^ Similarly, CRC-derived sEV miR-25-3p is transferred from cancer cells to endothelial cells (ECs), where it targets KLF2 and KLF4 and regulates the expression of VEGFR2, ZO-1, and Claudin5 proteins, thereby enhancing vascular permeability and promoting tumor angiogenesis.^83^ Additionally, TDSEVs carry angiogenic molecules such as miR-221-3p, miR-1246, miR-3157, and miR-210-3p, which activate signaling pathways in ECs to boost tumor vascular density and promote angiogenesis in various cancers, including cervical squamous cell carcinoma,^84^ non-small cell carcinoma,^85^ and oral cancer.^86^

In summary, experimental evidence confirms that an acidic TME enhances sEV release from cancer cells and results in the accumulation of sEVs within the tumor, which subsequently disseminate through the bloodstream, thereby promoting tumor growth and metastasis. Using this understanding for diagnostic and therapeutic purposes could represent a groundbreaking advancement in cancer treatment.

### 3.3. Immunosuppressive TME (iTME)

**Figure 4.**
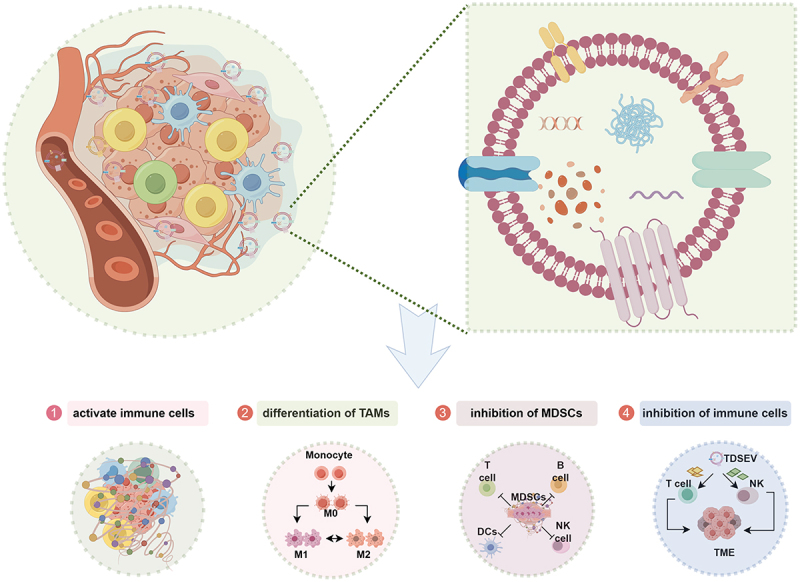
Regulation of immune cells in an immunosuppressive microenvironment by tumor-derived small extracellular vesicles (TDSEVs). ① TDSEVs activate immune cells and promote antitumor immunity by carrying tumor-associated antigens (TAA) in their cargo. ② TDSEVs play a crucial role in the conversion of M0 macrophages to M2 macrophages, commonly referred to as tumor-associated macrophages (TAMs). ③ TDSEVs enhance the inhibitory effect of myeloid-derived suppressor cells (MDSCs). ④ it has been observed that TDSEVs can inhibit the function of immune cells.

In contrast to other hypoxic, acidic, and nutrient-deprived microenvironments, there has been limited investigation into the regulatory mechanisms governing the release and cargo sorting of TDSEVs within the iTME. The impact of TDSEVs on immune cells within the TME is dualistic, as they can either stimulate or inhibit immune responses.^87,88^ TDSEVs can facilitate antigen presentation and thereby activate the immune system.^87^ Conversely, TDSEVs can also induce immunosuppression by delivering ligands, proteins, and miRNAs that inhibit immune cell activity.^89^ Presently, it is known that TDSEVs can regulate the TME by activating immune cells,^90^ promoting the differentiation of tumor-associated macrophages (TAMs),^91^ enhancing the inhibitory effect of TAMs,^91^ and inhibiting immune cell function, such as T cells,^92^ MDSCs,^73^ and NK cells^42^ (Figure 4).

#### 3.3.1. Activating immune cells

TDSEVs can activate immune cells and promote antitumor immunity by carrying tumor-associated antigens (TAAs) in their cargo.^93^ The presence of these antigens, along with major histocompatibility complex (MHC) molecules, suggests the potential development of TDSEVs as anticancer vaccines.^87^ Moreover, TDSEVs can deliver heat shock protein 70 (HSP70) and MHC class I molecules to dendritic cells (DCs), thereby inducing the activation of CD8^+^ T cells with the potential to exert an anticancer effect.^90,94^ Additionally, TDSEVs can create a localized inflammatory environment that enhances immune responses, making them a potent adjuvant in anticancer therapy.^95^ However, the immune-stimulating ability of TDSEVs may not always be sufficient to impede tumor progression, possibly due to the dual opposing roles of these vesicles within the TME.

#### 3.3.2. Promoting TAM differentiation

TAMs are polarized M2 macrophages that play essential roles in regulating the iTME.^96^ Studies have confirmed the important roles of TDSEVs and their cargos in mediating the communication between tumor cells and TAMs, thereby reprogramming the host immune response.^97^ Specifically, sEVs derived from breast cancer (BRCA) have been shown to induce M2 polarization of macrophages by down-regulating PTEN and activating the AKT/STAT3/6 signaling pathway, thereby promoting BRCA progression.^98^ Similarly, the polarization of M2 macrophages and the secretion of sEV LINC00273, facilitated by tumor-derived sEV miR-19b-3p, contribute to the metastasis of lung adenocarcinoma via the Hippo pathway.^99^ Moreover, the induction of M2 polarization in macrophages by tumor-derived sEV miR-934 promotes liver metastasis in CRC.^100^ Despite significant advancements in understanding the mechanisms by which TDSEVs promote TAM differentiation, the communication network between TDSEVs, tumor cells, and TAMs remains incompletely understood.^101^ Therefore, further elucidating the mechanisms of information transfer between TDSEVs, TAMs, and tumor cells, as well as their mechanisms of action, could facilitate the development of novel therapeutic strategies and their clinical applications.

#### 3.3.3. Enhancing the immunosuppression of MDSC

MSDCs are a subset of neutrophils and monocytes that are pathologically activated with immunosuppressive activity, serving as negative regulators of immunity. Currently, TDSEVs have been shown to play a crucial role in the immunosuppression of MDSCs. Over a decade ago, Xiang et al. demonstrated that TDSEVs can promote MDSCs to produce inhibitory molecules and enhance their inhibitory activity in tumor models.^102^ Subsequently, TDSEV proteins and nucleic acids have been identified as involved in regulating MDSC expansion and immunosuppression. For instance, mammary carcinoma-derived sEVs containing abundant prostaglandin E2 (PGE2) and transforming growth factor-beta (TGF-β) enhance the expansion and immunosuppression of MDSCs via the MyD88 pathway by increasing the production of interleukin-6 (IL-6) and vascular endothelial growth factor (VEGF).^103^ Glioma-derived sEV miR-1246 promotes MDSC differentiation and activation.^104^ Moreover, under hypoxic conditions, glioma cells secrete sEVs containing high levels of miR-29a and miR-92a, which can be transferred to MDSCs, thereby enhancing MDSC differentiation and function.^105^ A recent study by Shokati et al. discussed the mechanism by which MDSC-derived sEV miRNAs and tumor-derived sEV miRNAs can regulate antitumor immunity by modulating the interaction between tumor cells and MDSCs in the TME.^106^ Collectively, these studies highlight the importance of TDSEVs in MDSC cell biology, gradually revealing the regulatory mechanisms of TDSEVs on MDSCs and providing significant insights for the development of specific targetable therapeutic strategies for eliminating MDSC-induced immunosuppression.

#### 3.3.4. Inhibiting T cell function

Emerging evidence suggests that TDSEVs are major contributors to inducing T cell dysfunction. TDSEVs can suppress T cell antitumor immunity through various mechanisms, including inhibiting T cell proliferation and response, promoting regulatory T cell (Treg) expansion, and inducing T cell apoptosis and exhaustion.^107^ For instance, sEV TGF-β derived from breast cancer cell lines has been demonstrated to suppress T cell proliferation.^108^ Interestingly, TDSEVs from breast cancer cell lines are significantly enhanced by the hypoxic TME, potentially contributing to breast cancer therapy resistance.^109^ Moreover, pancreatic cancer-derived sEVs upregulate numerous genes associated with apoptosis and endoplasmic reticulum (ER) stress in T cells, and the uptake of these TDSEVs by T lymphocytes can trigger p38 MAP kinase signaling, leading to ER stress-induced apoptosis in T cells.^110^ In addition to their direct effects on T cells, TDSEVs also induce T cell inhibition by affecting DCs and MSDCs. For example, prostate cancer-derived sEVs impair DC antigen presentation, promoting adenosine-mediated inhibition of CD8^+^ T cell activation.^111^ Furthermore, melanoma-derived sEVs and TME accessory cells impair DC function, leading to the expansion of Tregs and MDSCs and limiting T cell cytotoxicity.^112^ While TDSEVs have been reported to affect T cell exhaustion,^107^ it remains largely unknown whether they also induce other states of T cell anergy, stemness, and senescence. Therefore, a deeper understanding of these molecular regulations will be crucial for the development of effective therapeutic strategies for cancer treatment.

#### 3.3.5. Inhibiting NK cell function

NK cells serve as the frontline defense against malignant cell transformation. However, tumor cells can undermine NK cell function through various mechanisms, with TDSEVs playing a pivotal role in inducing NK cell dysfunction. Multiple studies have indicated that TDSEVs can deliver their cargo to NK cells via cell membrane fusion, thus hindering their antitumor activity.^113^ TDSEVs employ six main strategies to inhibit NK cell function. Firstly, the uptake or interaction of TDSEVs with NK cells is believed to contribute to immune suppression and tumor evasion. For example, sEVs from pancreatic cancer cells (L3.6pl) and murine mammary carcinoma cells (TS/A) are internalized by NK cells and remain stably present in the cytoplasm, leading to decreased cytotoxic activity.^114,115^ Secondly, TDSEVs regulate NK cell migration and recruitment. Hong et al. demonstrated that sEVs isolated from patients with acute myeloid leukemia significantly reduced the migration of NK-92 cells toward tumor cells.^116^ Thirdly, TDSEVs influence NK cell proliferation and survival. According to Hong et al., sEVs derived from gastric cancer reduce the proliferation of NK-92 cells and decrease the frequency of CD8^+^ T and NK cells.^116^ By investigating the impact of TDSEVs on NK cell proliferation and survival, Liu et al. observed that pre-treatment with sEVs derived from murine breast carcinoma decreased the number and percentage of NK cells in vitro.^115^ Furthermore, TDSEVs modulate the cytolytic activity of NK cells. For instance, NK cells exposed to sEVs from pancreatic cancer display reduced cytotoxicity against pancreatic cancer stem cells.^114^ Although co-incubation of NK cells with oral cancer-derived sEVs initially enhances their killing effect on oral cancer cells, prolonged exposure leads to a significant decrease in cytotoxicity, suggesting that while TDSEVs may stimulate NK cell cytotoxicity in the short term, long-term exposure inhibits their cytolytic function, promoting immune evasion and cancer progression.^117^ Additionally, TDSEVs regulate cytokine production by NK cells. Cholangiocarcinoma-derived sEVs were found to significantly reduce the release of TNF-α by NK cells, impairing their ability to combat tumors.^118^ Similarly, NK cells exposed to sEVs from pancreatic cancer exhibited a notable decrease in TNF-1 and IFN-γ secretion.^114^ Moreover, TDSEVs alter the expression patterns of receptors and molecules in NK cells, potentially contributing to tumor-associated NK cell dysfunction. Since TDSEVs reflect parental cell contents, they likely play a role in modulating receptor and molecular expression, further influencing NK cell function.^119^ Recent research indicates that oral cancer-derived sEVs initially increase the expression of activating receptors on NK cells for 24 hours, but this effect diminishes over 7 days.^117^ Overall, studying the effects of TDSEVs on NK cells underscores the role of tumor-derived sEVs in immunosuppression. However, numerous other unknown or understudied TDSEV molecules contribute to NK cell dysfunction in tumors.^120^ Further research is needed to elucidate how TDSEVs affect NK cell function and improve cancer treatment.

### 3.4. Nutrient-deprived TME

The Warburg effect, a hallmark of tumor energy metabolism, is intricately regulated by the TME. In this nutrient-deficient milieu, characterized by high glucose consumption, recent research led by Kimryn Rathmell’s group revealed that myeloid cells display the highest capacity for glucose uptake within the tumor.^6^ Surprisingly, cancer cells exhibit a predominant uptake of glutamine,^6^ challenging the conventional idea of metabolic competition between cancer and immune cells in the TME. This discrepancy underscores the selective distribution of nutrients orchestrated by an intrinsic program within the TME.

#### 3.4.1. TDSEVs-mediated metabolic reprogramming

Metabolic reprogramming, a hallmark of cancer, allows cancer cells to thrive and proliferate within the nutrient-deprived TME, with TDSEVs emerging as pivotal mediators in this process.^121^ TDSEVs modulate the metabolism of recipient cells to satisfy energy and biosynthesis demands.^11^ Park et al. reported that sEVs derived from pancreatic cancer are enriched in adenosine diphosphate ribosylation factor 6 (Arf6), which enhances the glycolytic rate of cancer cells, thereby regulating the Warburg effect, fulfilling the energy and nutrient requisites of tumor cells, and facilitating tumor cell proliferation.^122^ Furthermore, TDSEVs play a crucial role in orchestrating the formation of premetastatic niches through metabolic reprogramming, thereby promoting tumor metastasis. Morrissey et al. demonstrated that TDSEVs induce the polarization of macrophages into an immunosuppressive phenotype by upregulating programmed death-ligand 1 (PD-L1) expression through NF-KB-dependent, glycolysis-dominated metabolic reprogramming, thereby establishing a premetastatic microenvironment.^123^ Recently, Li et al. uncovered a metabolic interplay involving sEVs-glutamine-fructose-6-phosphate amidotransferase 1 (GFAT1) from BCa and heightened angiogenic activity in ECs within the nutrient-deprived TME. They proposed inhibiting sEV-mediated GFAT1 secretion and targeting seryl-tRNA synthetase (SerRS) O-GlcNAcylation in ECs as potential strategies for antiangiogenic therapy in BCa.^124^ Moreover, TDSEVs contribute to metabolic reprogramming and remodeling of the microenvironment, exacerbating tumor angiogenesis and drug resistance. Among angiogenic factors such as proteins and nucleic acids, TDSEVs induce changes in ECs, augmenting their proliferation, migration, and angiogenic potential.^36,125^ Additionally, TDSEVs transferred from drug-resistant tumor cells to drug-sensitive counterparts confer increased resistance.^126,127^ Although TDSEVs mediate metabolic reprogramming that fuels tumor progression, studies on lipid and amino acid metabolism orchestrated by TDSEVs remain scarce. Thus, further investigation into lipid and amino acid metabolism is warranted to unravel how TDSEVs transport metabolites that foster tumor development.

#### 3.4.2. Indirect regulation of TDSEVs release

Within the hypoxic TME, lactate production is governed by the Warburg effect, which regulates glycolytic substrate phosphorylation and mitochondrial oxidative phosphorylation. Additionally, the acidic TME indirectly influences the release of TDSEVs. Pyruvate kinase M2 (PKM2), a crucial component of the Warburg effect in tumor cells, is overexpressed in the TME. Wei et al. concluded that PKM2, upon phosphorylation and dimerization, not only switches tumor cell metabolism from oxidative phosphorylation to aerobic glycolysis but also promotes tumor cell sEV secretion by directly phosphorylating synaptosomal-associated protein 23 (SNAP-23).^128^ The bidirectional metabolic reprogramming mediated by sEVs occurs between tumor cells and the TME. TDSEVs induce non-tumor cells in the TME to acquire a malignant phenotype, leading to the secretion of sEV cargo that regulates the metabolic reprogramming of tumor cells, thereby accelerating tumor progression and increasing TDSEV release and heterogeneity. Consequently, malignant positive feedback regulation patterns are established between the nutrient-deficient TME and tumor cells through TDSEVs.

## 4. Targeted TME and TDSEVs strategies

**Figure 5.**
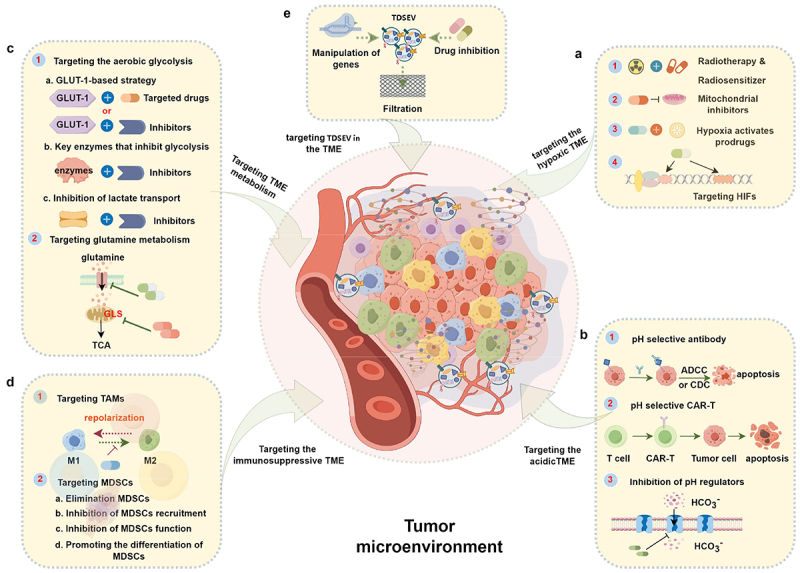
Therapeutic strategies based on targeting characteristics of the tumor microenvironment (TME) and tumor-derived small extracellular vesicles (TDSEVs). A. Targeting the hypoxic TME. These strategies mainly include the use of electron affinity radiosensitizers, enhancing tumor tissue oxygenation, hypoxia-activating prodrugs (HAPs), and targeting hypoxia-inducible factors (HIFs) and downstream targets. B. Targeting the acidic TME. This involves the development of acidic pH-selective antibodies, acidic pH-selective chimeric antigen receptor T (CAR-T) cells, and targeted inhibition of pH modulators. C. Targeting TME metabolism. Major strategies focus on aerobic glycolysis and glutamine metabolism pathways. D. Targeting the immunosuppressive TME. Strategies involve focusing on tumor-associated macrophages (TAMs) and myeloid-derived suppressor cells (MDSCs). E. Targeting TDSEVs in the TME. Major strategies include genetic manipulation, drug inhibition, and clearance of TDSEVs.

TDSEVs play a crucial role in shaping the TME and facilitating intercellular communication between cancerous and stem cells. This interaction ultimately promotes the maturation of the TME, leading to enhanced tumor growth and proliferation. Consequently, strategic targeting of TDSEVs and/or the TME presents a promising approach to prevent metastasis, overcome acquired resistance, and improve the overall efficacy of therapeutic interventions.^129,130^ Herein, we comprehensively outline the tumor’s hypoxic, acidic, metabolic and immunosuppressive microenvironment, along with the targeting strategies of TDSEVs, as illustrated in Figure 5.

### 4.1. Targeted hypoxic TME strategy

Hypoxia is a prevalent condition within solid TMEs, profoundly influencing tumor cell behavior such as proliferation, invasion, and metastasis. Additionally, it significantly diminishes the effectiveness of various treatment modalities like chemotherapy, radiotherapy, and photodynamic therapy. Therefore, targeting the hypoxic TME holds promise for enhancing antitumor effectiveness.

#### 4.1.1. Electron affinity radiosensitizer strategy

During the 1970s, it was shown that electron affinity compounds like metronidazole and Tirapazamine (TPZ), alongside hypoxia-activated cytotoxic drugs, could act as oxygen-independent radiosensitizers, enhancing the effectiveness of radiotherapy for hypoxic tumors. However, the heterogeneity of tumors and the uneven distribution of oxygen within them, especially in regions distant from blood vessels, present significant challenges for conventional hypoxia-activating drugs. As a result, sensitizing hypoxic tumors to radiotherapy remains a considerable challenge.^131,132^ In a recent study, Zhang et al. developed a multifunctional nanocapsular system incorporating cisplatin, metronidazole, and intracapsular encapsulated TPZ.^133^ This system demonstrated synergistic enhancement of radiosensitivity, a significant reduction in radiation dose, and substantial inhibition of tumor growth and metastasis under hypoxic conditions. Meanwhile, Xiao et al. designed multifunctional Au@AgBiS2 nanoparticles as highly effective radiosensitizers with significant antitumor immune activity, preventing tumor growth and lung metastasis.^134^ Moreover, Ma et al. utilized M1 macrophage-derived sEVs as a conceptual model to design effective radiosensitizers, achieving effective alleviation of tumor hypoxia, enhancement of DNA damage, inhibition of DNA damage repair, and ultimately remodeling the tumor suppressive microenvironment, leading to significant antitumor effects.^135^ The demonstrated efficacy of engineered immune cell-derived sEVs as radio-sensitive agents motivates future efforts to explore different types of immune cell-derived sEVs for enhanced radiotherapy.

#### 4.1.2. Enhancing oxygenation strategy

Starting from trials conducted in the 1960s exploring hyperbaric oxygen as a radiosensitizer, numerous clinical investigations have addressed tumor hypoxia by enhancing tissue oxygenation. In a recent study, Ashton et al. identified inhibitors targeting the mitochondrial electron transport chain (ETC) complex to impede oxygen consumption by tumor parenchyma.^136^ Skwarski et al. demonstrated that Atovarone, an FDA-approved mitochondrial inhibitor, effectively augmented tumor oxygen levels and suppressed hypoxic gene expression in individuals diagnosed with non-small-cell lung cancer (NSCLC).^137^ Additionally, Papaverine, an FDA-approved antispasmodic agent, and its derivatives have shown the ability to decrease mitochondrial oxygen consumption by inhibiting ETC complex *I* and alleviate tumor hypoxia in preclinical models.^138^ Furthermore, several nanoparticle platforms that enhance tumor oxygenation are undergoing preclinical development, including agents that carry or produce O_2_.^139,140^ Advances in these therapeutic techniques to improve their stability, biocompatibility, payload composition, and tumor targeting may enable drug and TME-modified therapies to be delivered to tumors. Although there have been no previous reports on the involvement of sEVs in tumor oxygenation strategies, recently Zhong et al. showed that ischemic limb-targeting sEVs derived from stem cells and oxygen-releasing nanoparticles can effectively treat critical limb ischemia in diabetic mice.^141^ This pioneering work suggests that co-delivery of sEVs and oxygen may be a promising strategy for tumor-targeted therapy.

#### 4.1.3. Hypoxia-activating prodrugs strategy

Hypoxia-activated prodrugs (HAPs) display selectivity in generating active anticancer agents under hypoxic conditions and have attracted significant attention in preclinical and clinical trials as primary drugs targeting hypoxia. Among these HAPs, TPZ and Evofosfamide have progressed to Phase III trials, showing promising clinical utility, although they have not yet received regulatory approval. Recently, Huang et al. achieved a remarkable antitumor rate of over 90% through the synergistic application of battery/HAPs.^142^ Additionally, Zhang et al. developed and synthesized a novel nanoparticle coated with TPZ as HAPs and zinc phthalocyanine (ZnPc) as a photosensitizer, demonstrating that this nanoparticle can activate HAPs effectively by photosensitive enhancement of hypoxia, thereby inhibiting glioma growth.^143^ These innovative approaches to synergistically sensitize and activate HAPs hold promise as potential strategies for tumor suppression and regulation of the TME.

#### 4.1.4. Targeted hypoxia-inducible factor strategy

Targeting hypoxia-inducible factors (HIFs) can disrupt their transcriptional regulatory function within the hypoxic microenvironment, thereby reducing the tolerance and adaptability of tumor cells to hypoxia. Pharmacological agents that target HIF and its downstream genes have shown significant efficacy in tumor suppression and have the potential to improve survival rates while exhibiting improved tolerability.^144^ Notably, studies have demonstrated that HIF-2α antagonists, such as PT2385 and PT2399, can inhibit the transcriptional activity of HIF-2, indicating strong antitumor properties.^145^ Belzutifan (PT2399), an inhibitor directly targeting HIF-2α, was shown in a phase III trial to be effective in renal cancer and other tumors in patients with von Hippel-Lindau syndrome,^146^ and it was recently approved by the FDA for this indication. However, these pharmaceutical agents may have specific toxicological and side effects, and some clinical trials lack sufficient data to comprehensively evaluate their safety and efficacy.^147^ Activation and inhibition of HIFs have become therapeutic targets for various cancers, with pharmacological approaches currently dominating the molecular strategy for targeting HIFs.^148^ In the future, sEV delivery systems based on precise targeting of HIFs are likely to be explored as an option.^149^

### 4.2. Targeted acidic TME strategy

The acidic pH observed in tumor environments stems from lactate production by tumor cells through the glycolytic pathway. This acidic pH creates a suppressive immune microenvironment, aiding tumor cells in evading immune detection and impacting the availability of classical antibodies. Currently, three primary strategies focus on targeting the acidic microenvironment of tumors.

#### 4.2.1. Development of acidic pH-selective antibodies

Sulea et al. have developed a pH-dependent HER2 antibody (bH1-P5P8) that exhibits enhanced antigen binding in acidic pH compared to neutral environments, leading to significant growth inhibition.^150^ Moreover, modifying the antibody Fc region to enable pH-dependent binding to C1q and FcγR can optimize the effector functions of antibody drugs, particularly antibody-dependent cell-mediated cytotoxicity (ADCC) and complement-dependent cytotoxicity (CDC).^151^ In a recent study, Li et al. successfully developed a tumor-selective, pH-dependent anti-CD47 antibody (BC31M4) that demonstrates both safety and potent efficacy in a xenograft solid tumor model.^152^ Additionally, Su et al. introduced a novel therapeutic approach involving the dual immune checkpoint blockade of CD47/PD-L1, specifically targeting the acidic microenvironment of tumors.^153^ This intervention effectively activates CD47/PD-L1, leading to a significant enhancement of the efficacy of CD47/PD-L1 antibody treatment for lung cancer.^153^ Despite being limited to preclinical stages at present, the application of pH-selective antibodies shows significant promise in improving the TME and addressing the challenges associated with the efficacy and safety of antibody therapy as a standalone treatment.

#### 4.2.2. Developing acidic pH-selective CAR-T therapies

The pH-dependent antibody scFv variable fragment functions as the extracellular targeting domain of chimeric antigen receptors (CARs), allowing for the selective delivery of CAR-T cells to the acidic TME. This targeted approach presents a potent strategy for reducing off-target toxicity. In a mouse model, a HER2-directed CAR incorporating a pH-restricted binding domain showed significant expansion of CAR-T cells and regression of HER2-positive tumor cells.^154^ However, despite the development of CAR-T products activated by acidic pH, most of these products remain in preclinical or clinical trial stages, and their safety profile continues to face significant challenges.

#### 4.2.3. Targeted inhibition of pH regulators

Acidification of the TME is essential for driving cancer progression as this influences the composition and function of stromal cells and provides favorable conditions for the survival and proliferation of malignant cells.^5,155^ Additionally, the acidic TME directly affects the effectiveness of immune checkpoint inhibitors.^156^ Therefore, targeting key pH regulators to prevent tumor acidification is of paramount significance. Mazzone et al. demonstrated that SLC4A4, highly expressed in pancreatic cancer, promotes the formation of an acidic TME through bicarbonate transportation. Importantly, targeted inhibition of SLC4A4 resulted in the reversal and reactivation of CD8^+^ T cell antitumor activity, improving immunosuppression and immunotherapy resistance.^157^

However, while the strategies targeting the acidic TME mentioned above have demonstrated a certain level of efficacy, their safety is yet to be fully clarified. Recently, multimodal targeted therapies based on the acidic TME have shown promising prospects for clinical translation. Tang et al. developed a pH-responsive AIE light-sensitive agent for the acidic TME, expected to demonstrate promising therapeutic effects on tumors while minimizing damage to normal tissues for precision photodynamic therapy.^158^ Gong et al. discovered that low-pH reprogrammed TDSEVs serve as a smart drug delivery platform, capable of specifically targeting tumor cells and selectively releasing various chemical drugs in response to sEV rupture caused by reactive oxygen species outbreak triggered by near-infrared irradiation. This approach demonstrates a safe and enhanced antitumor effect, with the potential for personalization for homologous tumors.^79^ These findings pave the way for novel avenues in sEV reprogramming.

### 4.3. Targeting TME metabolic strategy

The metabolic activities in tumors significantly influence essential biological processes such as cell proliferation, growth, migration, and invasion. Thus, it is imperative to target tumor cell metabolic pathways and enhance the nutritional environment of the TME to prevent tumor onset and progression. Herein, we discuss potential strategies aimed at disrupting aerobic glycolytic pathways and glutamine metabolism pathways that could be considered to achieve these objectives.

#### 4.3.1. Targeting the aerobic glycolysis pathway

The overexpression of glucose transporter 1 (GLUT-1) facilitates increased glucose uptake by tumor cells, leading to metabolic reprogramming and significant alterations in the TME.^159^ The increased glucose uptake facilitated by overexpressed GLUT-1 has led to the development of sugar binders capable of traversing GLUT-1 and delivering anticancer agents without inhibiting GLUT-1, emerging as a promising strategy for targeted antitumor therapy. Research in this domain has primarily focused on breast cancer, with Adriamycin,^160,161^ paclitaxel,^162^ and platinum compounds^163,164^ serving as the principal targeted drugs. Furthermore, STF-31, a small molecule inhibitor of GLUT1, has demonstrated notable antitumor effects.^165^ Subsequent investigations have revealed that STF-31 inhibits nicotinamide phosphoribosyltransferase (NAMPT), suggesting that GLUT1 is not the sole target of STF-31 inhibition.^166^ Another inhibitor of GLUT1, known as Glutor, targets GLUT1, GLUT2, and GLUT3 to impede glycolysis.^167^ BAY-876 represents the initial highly selective GLUT1 inhibitor.^168^ However, neither Glutor nor BAY-876 has exhibited in vivo antitumor efficacy,^167,168^ and whether they possess the requisite pharmacokinetic properties for clinical application remains to be determined. Hexokinase (HK) is the initial enzymatic step in the glycolytic pathway, and its isoform HK2 has been implicated in tumorigenesis.^169^ Various inhibitors, including 3-bromopyruvate,^170^ glucosamine derivatives,^171^ benitrobenrazide,^150^ and others, have demonstrated efficacy in mouse models by specifically targeting HK2.

Pyruvate kinase (PK), an essential enzyme in glycolysis, presents in three distinct isoforms: PKM1, PKM2, and PKLR. Inhibitors targeting PKM2 are believed to exert in vivo antitumor effects on xenografts derived from NSCLC by modulating biosynthesis and reducing the metabolic flux from glucose to lactate.^172^ However, it has been observed that PKM2 is not indispensable for tumorigenesis in specific models, prompting further investigation into the potential role of PKM2 inhibitors or activators, such as TEPP-46, in cancer therapy. Lactate dehydrogenase (LDH) exists in the form of LDHA and LDHB homotetramers and heterotetramers, which play a crucial role in the Warburg effect. In a mouse model of lung cancer, tumor suppression was achieved through the knockdown of LDHA.^173^ Despite the development of several potent LDHA inhibitors, selective inhibition of these inhibitors using small molecules has encountered limited success.^174–176^

Moreover, inhibiting monocarboxylate transporter 1 (MCT1) or MCT4, components of the SLC16A family responsible for lactate transportation, can lead to intracellular lactate accumulation and subsequent glycolysis suppression. Therefore, MCT1 or MCT4 inhibition demonstrates potential antitumor properties.^177,178^ Nevertheless, it is essential to acknowledge that these inhibitors can cause severe adverse effects, and their clinical effectiveness requires further validation through additional trials.

#### 4.3.2. Targeting glutamine metabolic pathways

The alanine-serine-cysteine transporter (ASCT2) facilitates active glutamine transportation into cells, where it is converted into glutamate through deamination by mitochondrial glutaminase GLS1 and GLS2 for metabolic purposes. The ASCT2 antagonist V-9302 has demonstrated antitumor properties and has also shown the ability to impede breast cancer progression by enhancing T-cell activation.^179^ Moreover, co-administration of V-9302 with the glutaminase inhibitor CB-839 led to a significant reduction in human HCC xenograft growth.^180^ Additionally, in line with V-9302‘s antitumor activity and its inhibition of L-type amino acid transporter 1 (LAT1)-dependent neutral amino acid transport, LAT1 was found to be essential for tumorigenesis in KRAS mutant CRC models.^181^

Thus far, numerous metabolic pathways have been targeted by key enzymes in cancer treatment. However, the variation in susceptibility of different tumor types to specific inhibitors warrants further investigation. Addressing the challenge of metabolic plasticity remains crucial for effectively targeting specific metabolic enzymes. To enhance safety and targeting precision, Nguyen et al. developed bioreducible sEVs that deliver acoustic sonosensitizers [triphenylphosphine coupled chlorine e6 (T-Ce6)] and glycolysis inhibitors (FX11) specifically to tumor mitochondria.^182^ Accumulation of T-Ce6 in mitochondria leads to their destruction upon exposure to ultrasound, resulting in accelerated cell death. Concurrently, F×11inhibits energy metabolism in cells, augmenting the efficacy of delivery therapies.^182^ This study introduces a novel approach to cancer treatment utilizing bi-stimulatory response sEVs to target hypoxic and metabolic pathways.

### 4.4. Targeting iTME strategy

Despite significant advances in cancer immunotherapy, immune checkpoint-targeting drugs are not yet universally available for cancer treatment. Instead, focusing on the immunosuppressive microenvironment of the tumor may hold the key to effective immunotherapy.^183,184^

#### 4.4.1. Targeting TAMs strategy

Currently, TAM-targeting drugs are classified into four main strategies. First, TAM removal focuses on the colony-stimulating factor 1 receptor (CSF1R) pathway, which regulates TAM function. CSF1R blockers and inhibitors can disrupt CSF1R signaling, effectively depleting TAMs, and have shown efficacy in preclinical models.^185–187^ Notable CSF1R blockers or inhibitors include Atezolizumab (RG7155), IMC-CS4, FPA008, and Pexidartinib (PLX3397).^188,189^ However, clinical use of these agents has led to serious adverse events, possibly due to off-tumor effects such as fatigue, weakness, anemia, nausea, facial and peripheral edema, lupus erythematosus, and hepatotoxicity.^190^ Secondly, inhibiting TAM recruitment involves disrupting the influx of TAMs by blocking circulating monocytes, which heavily rely on various chemokine signals.^191^ The CCL2/CCR2 signaling pathway regulates the recruitment of circulating monocytes into the TME, making it a promising target for TAM therapy.^192^ Inhibition of the CCL2/CCR2 signaling pathway has demonstrated antitumor effects in various experimental animal models.^193^ However, discontinuation of CCL2/CCR2 inhibitors can lead to a significant release of monocytes previously sequestered in the bone marrow, resulting in an overshoot of metastases and accelerated mortality.^194^ Therefore, alternative targets that address these limitations are crucial for designing future clinical trials aimed at achieving optimal and stable therapeutic responses. Thirdly, promoting TAM phagocytic activity involves inducing direct or indirect pro-inflammatory factors such as IFN, IL4, IL13, VEGF, GM-CSF, CSF-1, Ang-2, CCL2, and other chemokines to polarize TAMs toward an M1 phenotype and exert antitumor effects. The polarization of TAMs is influenced by the interactions between cytokines, chemokines, growth factors, and their receptors.^195^ This interaction can be utilized either independently or in combination with other strategies to enhance antitumor therapy.^196–198^ Modified TAMs can enhance the recruitment of cytotoxic lymphocytes and boost the tumor-killing capabilities of memory T cells.^197^ Fourthly, targeting TAM receptors (TAMR) represents a promising therapeutic approach for TAMs.^199^ TAMR comprises a family of receptor tyrosine kinases with a shared ligand, Gas6, and protein S, which drive macrophages toward a tumor-promoting M2-like phenotype.^199^ Thus, blocking TAMR signaling holds potential as an immunotherapy strategy. Small molecule inhibitors, antibody-drug conjugates (ADCs), chimeric antigen receptor T-cell therapy (CAR-T), and fusion proteins targeting TAMR are currently under development.^199^

#### 4.4.2. Targeting MDSC strategy

MDSCs present a significant obstacle to the effectiveness of immunotherapy across various cancer types, underscoring the importance of targeting them to enhance treatment outcomes.^200^ To this end, researchers are exploring several therapeutic avenues aimed at eradicating MDSCs or neutralizing their tumor-promoting activities. These approaches primarily fall into four categories. Initially, MDSCs were targeted using antibodies against surface markers like Gr-1 or Ly6G.^201^ Subsequent strategies have focused on more selective methods, such as S100A9-based “peptibodies”,^202^ apoptosis induction,^203^ and the use of chemotherapy drugs to clear MDSCs.^204^ Treatment employing these methods has shown promise in reducing MDSC numbers, leading to immune system recovery and tumor regression.^204,205^ However, clinical trials assessing the efficacy of these approaches have been limited, with inconsistent results regarding MDSC function and abundance.

Second, inhibiting the recruitment of MDSCs to the tumor site represents another potentially important strategy. Chemokine receptors play a pivotal role in facilitating MDSC migration, making blocking their interaction with ligands a logical approach to impede MDSC aggregation in the TME. Notably, targeting the interaction between the CCL2-CCR2 and CCL5-CCR5 axes has shown promising efficacy in combating tumor growth.^206–208^ Additionally, inhibiting CSF1R has demonstrated a reduction in MDSC recruitment. CSF1R inhibitors such as IMC-CS4, GW2580, PLX3397, and AMG820 have exhibited antitumor efficacy by impeding the survival of monocytic MDSCs (M-MDSCs) and TAMs.^209^ Several MDSC inhibitors, including rapamycin, AZD5069, Plexidartinib and Maraviroc, have been evaluated in clinical trials, particularly in phase I and II clinical trials.^210^

Third, reversing the immunosuppressive function of MDSCs represents another crucial strategy. Blocking the immunosuppressive mechanisms of MDSCs is fundamental for reinvigorating T cell activity and enabling successful immunotherapy. Phosphodiesterase-5 (PDE-5) inhibitors have been found to impede the function of MDSCs by reducing the expression and activity of iNOS and ARG1. Administration of PDE-5 inhibitors such as sildenafil and tadalafil in mouse models has been shown to reactivate the immune response against tumors through T cells and NK cells, leading to prolonged survival.^211^ Clinical trials have demonstrated enhanced intratumoral T-cell activity and improved outcomes in patients with head and neck squamous cell carcinoma (HNSCC) and metastatic melanoma.^212,213^ Additionally, targeting other molecules such as phosphatidylinositol 3-kinase (PI3K), Entinostat, and STAT3 inhibitors can also reverse the immunosuppressive function of MDSCs.^214–216^

Fourth, inducing the differentiation of MDSCs into non-suppressive cells could be another promising strategy. Administration of all-trans retinoic acid (ATRA) has been shown to accelerate the differentiation of MDSCs into mature myeloid cells, such as macrophages and DCs, thereby enhancing the T cell response in cancer patients.^217^ Additionally, vitamin D3 is another agent that has been reported to promote the maturation of myeloid cells and reduce the number of MDSCs in cancer patients.^218^

While all four of the above strategies targeting MDSCs have shown promising advances in preclinical applications, two major challenges hinder their translation to the clinic. First, the heterogeneity of MDSCs poses a significant obstacle to their targeted therapies.^219^ The immunophenotypes and mechanisms of MDSCs vary across different tumor types and even within the blood and tumor tissues of the same patient. Isolating and purifying MDSCs from the TME is challenging, and distinguishing between MDSCs and normal bone marrow stem cells is unclear, limiting specific detection and targeted therapy of MDSCs in cancer.^220^ Second, the lack of molecular studies on the differentiation process and mechanism of action of MDSCs has impeded the development of antitumor therapeutics targeting MDSCs.^221^ In the future, further understanding of the specific mechanisms of MDSCs in tumor occurrence and development, as well as their participation in the formation and evolution of polymorphonuclear MSDCs (PMN-MDSCs), is necessary to improve the verification and detection of MDSC phenotypes. This understanding will be conducive to enhancing targeted therapy strategies for MDSCs.

### 4.5. Inhibition strategies for TDSEVs

Emerging novel strategies, such as genetic manipulation, pharmacological inhibition, and TDSEV clearance, are being developed to selectively inhibit TDSEVs by targeting their biological production and release mechanism.

#### 4.5.1. Genetic manipulation strategies

Prominent biotechnological tools, such as RNA interference (RNAi) and CRISPR-Cas9 systems, have been extensively used to attenuate or disrupt the expression of essential genes involved in TDSEV biogenesis and secretion.^222^ The ESCRT pathway, pivotal in the mechanism of TDSEV biogenesis, plays an important role in this process. Using RNAi, a research team from Colombo silenced ESCRT-related components (HRS, STAM1, TSG101, etc.) in HeLa cells, leading to reduced secretion of TDSEVs and sEV MHC class II.^223^ Similarly, Hoshino et al. knocked down HRS in SCC61 HNSCC cells, which significantly decreased the secretion of sEVs and proteins.^224^ Disruption of HRS expression by RNAi has also been shown to markedly reduce sEV PD-L1 levels.^92^ Consequently, targeting HRS holds promise for efficiently suppressing both TDSEVs and cargo.

Additionally, inhibition of TDSEVs has been reported to target Rab GTPases involved in intracellular vesicle transport. RNAi-mediated gene knockdowns of Rab27a or Rab27b have been reported to inhibit sEV secretion in various tumor cells, including BCa cells,^225^ HNSCCs,^224,226^ and cervical cancer cells.^39^ Similarly, Poggio et al. used CRISPR-Cas9 to knock out nSMase2 or Rab27a, achieving inhibition of sEV secretion in prostate cancer cells.^227^ However, deleting nSMase2 only partially eliminated TDSEVs, indicating the need for further investigation into more effective strategies or targets. Additionally, RNAi targeting Rab7 in MCF-7 cells inhibited MVB transport and resulted in reduced TDSEV secretion.^228^ Recently, it was shown that Rab31 regulates sEV biogenesis in HeLa cells through an ESCRT-independent pathway and drives cargo sorting, offering novel targets for TDSEV and cargo inhibition in future therapies.^229^ For SNARE proteins (i.e., syntaxin 6, VAMP7, and YKT6), which mediate the fusion of MVB with the cell membrane, down-regulation of syntaxin 6 expression significantly reduced TDSEV secretion by prostate cancer cells.^230^ Furthermore, Ruiz-Martinez et al. found YKT6 inhibition of TDSEV secretion in A549 cells.^231^ TDSEV inhibition strategies based on genetic manipulation have promising applications in cancer research, but they still face many challenges.^232^ First, off-target effects can reduce blocking effects and raise safety concerns.^233^ Second, the specificity of current inhibition strategies is limited, as blocking TDSEVs also blocks non-TDSEV secretion, potentially inducing side effects in tumor therapy. Third, biosafety concerns may arise with genetic modification based on viral systems.^234^ Therefore, a comprehensive understanding of TDSEV biogenesis and secretion is needed to identify more potential targets for future translation. Moreover, efforts should focus on developing multi-target strategies to inhibit each critical step in TDSEV generation, leading to a whole pathway of TDSEV inhibition.^227^

#### 4.5.2. Pharmacological inhibition strategies

Pharmacological inhibitors of TDSEVs have been extensively studied in recent decades and have shown great promise for therapeutic applications. Currently, pharmacological inhibition strategies can be divided into three categories, namely, TDSEV inhibitors, specific inhibition of genomic mutations, and shared regulatory mechanisms.

TDSEV inhibitors can be used to inhibit TDSEV biogenesis and secretion mechanisms. Among these inhibitors, GW4869 is widely recognized as the most frequently employed. In both in vitro and in vivo experiments, GW4869 inhibited sEV secretion from various tumor cells, including breast cancer cells,^235^ epidermal cancer cells,^236^ BCa cells,^225^ HNSCC cells,^224^ and malignant melanoma cells,^237^ thereby promoting antitumor immunity.^235,238–240^ Yang et al. found that GW4869 inhibited TDSEV secretion and reduced the total protein content in breast cancer cells.^235^ Moreover, in a mouse model of breast cancer, GW4869 suppressed tumor growth by inhibiting TDSEV, thereby promoting antitumor effects and significantly enhancing the therapeutic effect of the anti-PD-L1 antibody.^235^ Wang et al. used hyaluronic acid to assemble nano-units of GW4869 and a ferro-death inducer (Fe^3+^), and the collaboration of the two active ingredients induced an antitumor immune response against B16F10 melanoma cells and stimulated cytotoxic T lymphocytes and immune memory.^238^ Although GW4869-based inhibition strategies have been reported in a large number of cases, several drawbacks limit their practical application. First, GW4869 is an nSMase2 blocker that mediates the biogenesis and secretion of TDSEV and non-TDSEV in an ESCRT-independent pathway.^240^ Thus, direct application of GW4896 without targeted delivery may result in nonspecific inhibition of non-TDSEV. Second, GW4869 removes nSMase in a noncompetitive manner, which may lead to less efficient TDSEV inhibition.^239^ Lastly, nSMase2 has been shown to be involved in multiple important biological processes,^241^ and GW4869 efficacy should be assessed in terms of biosafety before clinical application.

Genomic mutations that cause aberrant biogenesis and secretion of TDSEVs can also be pharmacologically inhibited. The RAS/RAF/ERK signaling pathway plays an essential role in intracellular and intercellular communication processes. The abnormal activation of the RAS signaling pathway is closely related to tumorigenesis. Farnesyltransferases (FTases) are the key enzymes for RAS protein activation. Thus, FTase inhibitors are antitumor drugs that target the Ras protein.^242^ It has been found that the FTase inhibitors manumycin-A and Tipifarnib lead to the inhibition of TDSEV secretion by prostate cancer cells by selectively affecting the RAS/RAF/ERK pathway.^243,244^ Recently, Sasabe et al. found that EGFR inhibitors inhibit the malignant potential of oral squamous cell carcinoma (OSCC) cells by directly inhibiting EGFR downstream signaling pathways as well as by inhibiting the uptake of TDSEVs through macropinocytosis,^245^ which confirms that anti-EGFR agents may be TDSEV inhibitors. Ketoconazole (KTZ) has been shown to inhibit pathways of sEV biogenesis and secretion.^246^ Greenberg et al. provided the latest evidence for the use of TDSEV inhibitors as a novel option for cancer treatment by enhancing the efficacy of sunitinib by adding KTZ to cause TDSEVs inhibition and reduce tumor proliferation in sunitinib-resistant renal cancer cells (786-O).^247^

Additionally, targeting the shared regulatory mechanisms and inhibition of the TME or genomic mutations could be considered. Given the observed over-expression and/or over-activation of key regulators in tumor cells, these molecules represent promising candidates for TDSEV inhibition.^248,249^ Syntenin-Syndecan-ALIX plays a key role in the biogenesis of EVs.^228^ It has been established that heparan sulfate analogs specifically and effectively inhibit the secretion of TDSEVs by B16F10 melanoma cells via targeted Syndecan-Syntenin-Alix, resulting in attenuated tumor proliferation and invasion.^248^ Im et al. used sulfamisoxazole (SFX) to selectively inhibit the transcription of Rab GTPases (Rab5, Rab7, and Rab27a) and ESCRT components (Alix, VPS4B), which inhibited multivesicular body (MVB) formation and secretion and ultimately resulted in the inhibition of TDSEV secretion.^250^

However, pharmacological inhibition strategies are limited in terms of the complexity of TDSEV biosynthesis and secretion pathways, requiring the development of multi-target inhibitors to effectively block TDSEV production. Moreover, the diversity of circulating sEVs presents challenges in selectively inhibiting TDSEV, thus requiring careful targeting to avoid affecting non-TDSEVs.^251^ Exploring modifications to the TMEs or targeting relevant signaling pathways may provide novel avenues for TDSEV inhibition. Additionally, the development of pharmacological inhibitors is a lengthy process and costly.^252,253^ Nevertheless, implementing a high-throughput screening system for TDSEV inhibitors, coupled with quantitative assays to simultaneously assess multiple candidate inhibitors, might enhance effectiveness.^243,244,250^

#### 4.5.3. Clearance strategies for TDSEVs

Orme et al. introduced therapeutic plasma exchange as a method to eliminate TDSEVs from the bloodstream in patients diagnosed with malignant melanoma.^254^ Dialysis, another popular method for removing detrimental substances from the circulatory system, utilizes semi-permeable membranes with diameters smaller than 1 nm to effectively remove toxicants. Microporous membranes of suitable size are anticipated to be employed for TDSEV removal. Wu et al. utilized silica microspheres and hemofiltration devices to capture and eliminate a substantial quantity of circulating tumor cells and TDSEV, presenting a promising tumor treatment avenue.^255^ The use of microfluidic chips, which have excellent compatibility and minute dimensions, allows the integration of numerous antibody-coated units to efficiently eradicate TDSEV, resulting in rapid and direct elimination.^256^

Current strategies for removing TDSEV using ex vivo devices have limitations such as trauma, bleeding, and infection risks. Additionally, dialysis can remove both harmful and beneficial sEVs, further burdening cancer patients. Future strategies should aim to be more specific and gentle.^254,257^ There is evidence suggesting that the role of phagocytic clearance by macrophages may be altered in the TME.^258^ High PD-1 levels in TME macrophages hinder phagocytosis, but blocking PD-1 could potentially boost TDSEV clearance. In the case of HNSCC, TDSEVs inhibit phagocytosis through CD73, thereby promoting tumor growth.^259^ Therefore, activating macrophage phagocytosis in vivo may prove more effective than creating a clearance system in vitro.

## 5. Summary and prospect

The TME plays an essential role in tumor development, invasion, and metastasis. Stress conditions such as hypoxia, starvation, and acidosis can induce heterogeneous changes in the release of sEVs by tumor cells, affecting cargo sorting and transport, thereby contributing to malignant proliferation, metastasis, and tumor resistance to treatment. TDSEVs activate immune cells and drive antitumor immunity through TAAs in their cargo. Furthermore, the antigens and MHC molecules they carry enable the development of anticancer vaccines. However, the ability of TDSEVs to stimulate the immune system and thus prevent tumor progression remains challenging. While strategies targeting the inhibition and removal of TDSEVs have shown promise in cancer treatment, the highly heterogeneous physical characteristics and molecular composition of TDSEVs within the TME remain bottlenecks for clinical translation. Off-target effects and biological safety risks limit gene manipulation in TDSEV inhibition for clinical applications. Therapeutic strategies targeting the TME have demonstrated beneficial antitumor effects in preclinical studies. However, while remodeling the TME improves targeting efficiency, it also increases the risk of tumor migration and metastasis. Tumor heterogeneity and complexity pose major challenges for TME remodeling. In recent years, sEVs have emerged as promising vehicles for drug delivery. Compared to conventional nanocarriers, sEVs offer advantages in terms of immunogenicity, in vivo barrier crossing, and drug metabolism, presenting a promising avenue for tumor-targeted therapy. Therefore, harnessing the information transmission properties of EVs, enhancing TME targeting efficiency and improving susceptibility to immunotherapy offer a potential strategy for developing effective and safe delivery systems based on TDSEVs and/or TME modulation. Collectively, this multimodal approach could hold promising potential for overcoming resistance to tumor immunotherapy in the future.
